# Quality in Standardized Patient Training and Delivery: Retrospective Documentary Analysis of Trainer and Instructor Feedback

**DOI:** 10.7759/cureus.21022

**Published:** 2022-01-07

**Authors:** Derya Uzelli Yılmaz, Nicole Last, Janice Harvey, Leigh Norman, Sandra Monteiro, Matthew Sibbald

**Affiliations:** 1 Department of Fundamentals of Nursing, Izmir Katip Celebi University, Izmir, TUR; 2 Centre for Simulation-Based Learning, McMaster University, Hamilton, CAN; 3 Faculty of Health Sciences, McMaster University, Hamilton, CAN; 4 Medicine, McMaster University, Hamilton, CAN

**Keywords:** quality improvement, quality assurance, standardized patient program, training, standardized patient

## Abstract

Background

An important aspect of developing and maintaining a high-quality standardized patient (SP) program is incorporating quality assurance processes. Trainer and instructor feedbacks are considered critical in achieving these goals. The aim of this study is to determine programmatic and systematic issues in the scope of quality assurance and improvement through trainer and instructor feedback on SP performance. We also presented a logic model based on a synthesis of the current literature to ensure the development and maintenance of a quality management culture in the SP program.

Methods

A retrospective analysis of SP scoring was conducted, and written feedback forms completed by trainers and instructors in a large Canadian university’s SP program were collected. The previous six years (2014-2020) of SP feedback forms in the scope of quality assurance were reviewed and analyzed. Descriptive statistics were utilized to analyze the ratings. Thematic analysis was conducted on the data gathered from the written feedback.

Results

A total of 138 feedback forms were reviewed and analyzed in the study. The mean ratings given by the trainers for feedback and professionalism were 4.27 ± 1.29 and 4.77 ± 0.8, respectively. The mean ratings given by the instructors for knowledge of case information, appropriate responses, and affect were 4.84 ± 0.64, 4.86 ± 0.35, and 4.71 ± 0.76, respectively (from a range of 1 to 5). Four key themes emerged from the written feedback: nonverbal behaviors in simulation activity or feedback sessions, providing feedback from the patient perspective, consistency between role portrayal and scenario, and adapting easily to changing situations.

Conclusions

Component scoring on SP performance did not discriminate individual issues, but the qualitative comments identified certain specific issues. Further research is needed to establish standards of continuous quality improvement (CQI) within an SP program.

## Introduction

Standardized patients (SPs), individuals who are specially trained to act as patients, are an effective teaching strategy employed for the purpose of improving medical students’ clinical and communication skills [1]. SP encounters help trainees develop clinical skills through the representation of diverse healthcare issues, including both physiological and psychological findings that help promote consistency, realism, and authenticity in clinical training programs [2]. In addition, licensing and specialty boards for healthcare disciplines make use of SP in the certification of certain clinical competencies [3]. SP programs are used increasingly by health sciences education programs as part of their curricular mandate to support the teaching and assessment of clinical skills. The Association of Standardized Patient Educators (ASPE) advocates for the implementation of quality assessment processes in SP education in order to deliver and sustain consistently high-quality SP-based education [4]. The central importance of quality assurance was highlighted by Nestel et al., who stated that a quality improvement culture within SP programs is essential for the growth, integrity, and safe application of SP-based education [5].

Performance evaluation of SPs is one of the indicators used in the quality assessment of SP training and delivery. While there are several models for the evaluation of an SP’s performance (e.g., the “Plan-Do-Study-Act Model” and the “Higher Learning Commission’s Assessment Culture Matrix to guide CQI efforts”), a common quality assurance method is post-assessment and analysis of live or recorded interactions [6-9]. Nestel et al. conducted an international comparative case study that identified key challenges in SP programs from Australia, Canada, Switzerland, and the United Kingdom, in that each program had informal measures in place to identify underperforming SPs [10]. Most programs assessed SP performance using a tutor and learner feedback, whereas a few SP programs used a standardized form in order to ensure some level of consistency in SP ratings [11]. Cantillon et al. reported on SP program development in medical education across four countries in Europe; however, their survey-based study identified a lack of consistency in quality assurance approaches for SP role portrayal [12].

**Figure 1:**
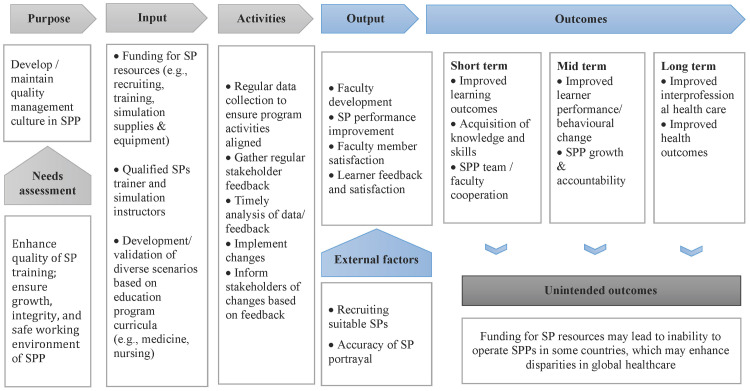
Example of a logic model to develop quality management culture in standardized patient program

Training, monitoring, and assessing SPs on a constant basis are required for timely and effective quality assurance [13]. SP performance is often influenced by the various individual interpretations of the quality assurance processes. Therefore, the focus of any SP assessment or feedback needs to be carefully considered and clearly framed. Although significant literature exists regarding the role of a quality improvement context in the strengthening of established SP programs, little data has been published that addresses the quality assurance activities in the scope of the SP’s actual performance. The aim of the current study is to determine programmatic and systematic issues in the scope of quality assurance and improvement through trainer and instructor feedback specifically on SP performance. Furthermore, the present study proposes an example of a logic model based on a synthesis of the current literature aimed at developing and maintaining a quality management culture in SP programs (Figure 1).

## Materials and methods

This retrospective study was conducted within a large Canadian university SP program that supports multiple healthcare training programs, including undergraduate and postgraduate medicine, physician assistant, undergraduate nursing and nursing assistant, midwifery, rehabilitative sciences, undergraduate health sciences, social work, continuing professional development, and licensure examinations. On average, the program provides approximately 10,000 hours of curricular support annually, with 200+ SPs and four SP trainers. SPs provide both faculty members and students opportunities to teach, assess, and refine a variety of skills, including communication, interviewing, diagnostic, and clinical skills. The current practice for evaluating SPs within the study setting is to collect feedback from all stakeholders (i.e., instructors, trainers, SPs, and learners). Feedback on SP interactions with learners is recorded using a structured feedback form; the trainers then meet with the SPs to review the written reports. This process helps SPs to better understand their role and how they can improve in the future to better support learning. Additionally, the present study proposes an example of a logic model based on a synthesis of the current literature aimed at developing and maintaining a quality management culture in SP programs (Figure 1).

The previous six years (2014-2020) of SP feedback forms were reviewed and analyzed. The trainers and instructors filled out feedback forms for SPs to ensure a high-quality SP program. The forms had each been completed by trainers or instructors for the purposes of quality assurance. Each form consists of both Likert-type and open-ended questions to elicit feedback. A Likert-type scale is a measure of attitudes designed to allow respondents to indicate how strongly they agree or disagree with carefully constructed statements and generally range from very negative to very positive toward a certain attitude or construct [14]. Both trainers and instructors rated SP performance on portrayal, case content, and the environment based on a scale ranging from “1” (needs to be retrained) to “5” (excellent).

Descriptive statistics were utilized using the program IBM SPSS Statistics version 22.0 (IBM Corp., IBM SPSS Statistics for Windows, Armonk, NY, USA) to analyze the ratings given by the trainers and faculty instructors in their feedback. Continuous variables are presented as median (minimum-maximum), while categorical variables are described using frequencies and percentages.

Qualitative analysis of the open-ended responses was conducted according to thematic analysis [15]. The authors (DUY, NL, and MS) conducted a simple conventional content analysis of each form and then thematically categorized each study by the scope of focus. Any discrepancies were discussed with at least one other reviewer until a resolution was reached.

The study was deemed exempt from ethical review by the Hamilton Integrated Research Ethics Board on March 23, 2020. It was not deemed possible to acquire consent from those participants who completed the feedback forms, because all data were anonymized to its source.

## Results

Results of quantitative data analysis

The trainers’ feedback regarding SP encounters (n = 87) were consisted of bachelor studies in health sciences (n = 48, 55.2%), nursing (n = 20, 23%), midwifery (n = 1, 1.1%), postgraduate health sciences (n = 14, 16.1%), and physician assistants (n = 4, 4.6%). Descriptive analyses of the feedback are presented in Table 1.

**Table 1:** Descriptive analyses of feedback on standardized patient encounters by trainers and instructors SPs: standardized patients

Descriptive characteristics	Trainers (n = 87) (n (%))	Instructors (n = 51) (n (%))
Educational program		
Bachelor of health science	48 (55.2)	1 (2)
Nursing	20 (23)	10 (19.6)
Postgraduate	14 (16.1)	2 (3.9)
Physician assistant	4 (4.6)	0 (0)
Midwifery	1 (1.1)	0 (0)
Medicine	0 (0)	6 (11.8)
Social work	0 (0)	9 (17.6)
Physiotherapy	0 (0)	4 (7.8)
Occupational therapy	0 (0)	19 (37.3)
Application of SPs		
History taking/interview	82 (94.3)	43 (84.3)
Physical examination	2 (2.3)	3 (5.9)
Both	3 (3.4)	5 (9.8)

The faculty instructors’ feedback on SP encounters (n = 51) were also consisted of bachelor studies in health sciences (n = 1, 2%), nursing (n = 10, 19.7%), medicine (n = 6, 11%), social work (n = 9, 17.8%), physiotherapy (n = 4, 7.8%), occupational therapy (n = 19, 37.3%), and postgraduate health sciences (n = 2, 4%).

While 94.3% (n = 82) of SP encounters upon which feedback was given by the trainers (n = 87) were made regarding patient history taking/interview, this figure was 84.3% (n = 43) for faculty instructors (n = 51).

The mean values of the “quality of SP,” “SP portrayal,” and “quality of scenario,” which were rated by the trainers, are presented in Table 2. While the quality of SP and SP’s portrayal was rated between 1 and 5, the quality of the scenario was rated between 1 and 3 by the trainers. The mean ratings for the feedback given by SP and the professionalism under the quality of SP were 4.27 ± 1.29 and 4.77 ± 0.81, respectively. Knowledge of case information, appropriate responses, and affect under the SP’s portrayal had a mean rating of 4.84 ± 0.64, 4.86 ± 0.35, and 4.71 ± 0.76, respectively. While the mean rating for the adequacy of case details was 2.91 ± 0.32, the mean rating for the scenario’s suitability and use of equipment was 2.87 ± 0.34.

**Table 2:** Mean of trainers’ feedback on the standardized patients SP: standardized patient; SD: standard deviation; min: minimum; max: maximum

Trainers’ feedback on SP	Mean ± SD (min–max)
Quality of SP	
Feedback given by SP	4.27 ± 1.29 (1–5)
Professionalism (e.g., preparedness and punctuality)	4.77 ± 0.81 (1–5)
SP’s portrayal	
Knowledge of case information	4.84 ± 0.64 (1–5)
Appropriate responses	4.86 ± 0.35 (4–5)
Affect (e.g., experience and consistency)	4.71 ± 0.76 (1–5)
Quality of scenario	
Adequacy of case details	2.91 ± 0.32 (1–3)
Suitability and use of equipment	2.87 ± 0.34 (2–3)

The mean rating for the “quality of SP,” “SP’s portrayal,” and “quality of scenario,” as given by the instructors, are presented in Table 3. The domains were rated between 1 and 5 by the instructors. The mean ratings for the feedback given by SP and the professionalism under the quality of SP were 4.77 ± 0.67 and 4.90 ± 0.45, respectively. Realism under the SP’s portrayal had a mean rating of 4.83 ± 0.51. While the mean rating for the adequacy of case details was 2.91 ± 0.32, the mean rating for the scenario’s suitability and use of equipment was 2.87 ± 0.34.

**Table 3:** Mean of Instructors’ feedback on standardized patients SP: standardized patient; SD: standard deviation; min: minimum: max: maximum

Instructors’ feedback on SP	Mean ± SD (min–max)
Quality of SP	
Feedback given by SP	4.77 ± 0.67 (1–5)
Professionalism (e.g., preparedness and punctuality)	4.90 ± 0.45 (2–5)
SP’s portrayal	
Realism (e.g., affect and appropriate responses)	4.83 ± 0.51 (2–5)
Quality of scenario	
Adequacy of case details	4.10 ± 0.46 (2–5)
Inaccuracies in case context	2.87 ± 0.34 (1–5)

Results of qualitative data analysis

Four key themes were constructed from the trainers’ and instructors’ written feedback regarding the quality of SP’s application.

Theme 1: Nonverbal Behaviors in Simulation Activity or Feedback Sessions

Much of the trainer feedback commented on the SP’s nonverbal behaviors (e.g., gestures and voice tonality) used during simulation activity or feedback sessions. While some of the trainer data reported that SP eye contact was a means for maintaining communication during the feedback/simulation, they also noted that the SP’s tone or speech was not necessarily matched to the case.

*Theme 2: Providing Feedback From the Patient’*​​​​​​*​s Perspective*

Both trainers and instructors commented on the importance of SPs’ feedback relating to how it made them feel or think as a patient versus medical content. SPs that gave feedback from the patient’s perspective were specifically mentioned. Some of the trainer and instructor data also recognized the need to provide feedback that is specific to what happened during the simulation. Therefore, it was noted that SPs could benefit from further support in the area of feedback practices.

Theme 3: Consistency With Role Portrayal and Scenario

Another issue addressed by both trainers and instructors was that SPs sometimes portray their role differently from the given scenario they are tasked with representing. Trainer data specifically pointed out that the SP’s portrayal depends on the characteristics of the learner group (professional discipline or level of experience) and the purpose of the learning activity, including learning objectives.

Theme 4: Adapting Easily to Changing Situations

Another point made clear in both the trainer and instructor data was that SPs must be able to adapt easily to changing situations (e.g., some questions not in the scenario raised by learners, to help learners to be more comfortable) within the session. This issue relates to unexpected questions or situations, and how much and when to give information to SPs.

Examples of quotes from trainers and instructors under the four themes of responses are shown in Table 4.

**Table 4:** Example quotes from trainers and instructors

Themes	Example quotes
Nonverbal behaviors in simulation activity or feedback sessions	“Maintained poor eye contact and was pacing. Answered questions clearly instead of pushing agenda…” (Trainer)
	“Could perhaps present as a bit more uptight, uncomfortable, and stressed with more tense body language and a worried demeanor. Also needed, add a bit of insecurity and lack of confidence to the characterization.” (Trainer)
	“Good affect and responses given to learners. One thing I would suggest is to remember to change your tone when talking about being angry/frustrated; you said, ‘I’m angry’ a few times, but your voice and tone remained the same (soft-spoken) – don’t be afraid to raise your voice a little bit!” (Trainer)
	“Slow, low-toned speech and would look down a lot, almost appears down in mood (not specific to the case), maybe even slightly confused.” (Trainer)
Providing feedback from the patient’s perspective	“Target the feedback a bit more to how it made the patient feel or think. For patient history, focus more on feelings avoided in life.” (Trainer)
	“The SP provided very specific and helpful feedback based on her experience as ‘Roberta’; i.e. she did not comment on the students’ occupational therapy skills and knowledge but helped them understand how it felt for her as the patient and provided excellent suggestions on what might have helped at the moment.” (Instructor)
	“Be more specific with feedback – put out a particular behavior that the student displayed that kept you calm.” (Trainer)
	“Feedback was not specific to the simulation. Asked the students to self-reflect and discussed what the students should have touched on. Not the patient perspective at all.” (Trainer)
	“For feedback, it was generally helpful and constructive, but one comment was ‘you did not demonstrate empathy,’ which could have been reworded.” (Instructor)
Consistency with role portrayal and scenario	“Some case information (where the character is living) was drastically different than the written case. Need to follow up with SP to see if they received some redirection. Gave out case information far too easily. The interviewer even stated, ‘he’s really chatty.’” (Trainer)
	“Overall tone was not in keeping with the character as written. The confrontational aspect and somewhat prejudicial attitude were lacking throughout.” (Trainer)
	“SP did not appear to be short of breath or wince with pain while coughing. Began to use body movements to indicate labored breathing only during the physical examination.” (Trainer)
	“Anger seemed to be scaled to the learner’s ability; potentially could have appeared to be more angry.” (Instructor)
	“Occasionally was a bit generous with giving out information, but given the junior level of the learner, this approach kept the interview moving.” (Trainer)
	“The SP was great! Met needs of this group of students.” (Instructor)
Adapting easily to changing situations	“A few unexpected questions came up (‘Why are you speaking to strangers, but not friends and family?’). These were well handled.” (Trainer)
	“Great affect! Was being interviewed by a very talkative learner and was very patient in not giving out information and allowing the learner to dictate the pace.” (Trainer)
	“Respond to questions wife (in the case) sufficient detail to cue student, but not so much to not refuse. Excellent!” (Instructor)

## Discussion

The present study explored programmatic and systematic issues with SP training and delivery through descriptive and thematic analyses based on trainer and instructor feedback. In total, the previous six years of SP scores were analyzed, which presented high overall evaluation levels of the SPs’ performance according to the trainers and instructors. However, it appears that component scoring did not discriminate against specific issues very well, and it is possible that the quality of SP performances may be adjusted psychometrically for scoring purposes. Other studies have also commented on the performance of SPs in providing information on SP program quality assurance scoring levels [8,16]. According to the scoring, SPs are routinely assessed and retrained to ensure that they are consistent and remain accurate in terms of role portrayal. For existing SP programs, scoring may also serve as a periodic evaluation tool from which to chart progress over a certain period of time. However, recognition of an acceptable level of SP performance may be a function associated with the standardization of quality processes [17].

The qualitative comments identified specific issues, with key themes constructed based on the value of human interaction in SP encounters. For example, many of the trainer and instructor comments are related to the SP’s nonverbal behaviors. SPs attempt to offer a realistic portrayal of a set character, as well as try to respond appropriately and constructively to questions posed by learners. One of the benefits of working with SPs is that they are able to provide feedback according to the patients’ perspectives [18]. Through careful observation of the learner’s behavior and close monitoring of the effects this behavior may have on the character’s feelings, appropriate feedback can be provided [19]. The trainers and instructors also noted that SP feedback must specifically relate to how something the learner said or did made the patient “feel or think.” SPs can focus on the actions and demeanors of the student and thereby shape their responses according to the patient case being portrayed. SPs need to be informed about their expected responses, as well as the actions that will be simulated throughout the scenario. In preparing for a specific role, SPs should be encouraged to think about unexpected questions in which the scenario could be varied [17,20].

Qualitative themes could be particularly used to construct quality assurance forms in an evidence-informed manner. Trainers, SPs, and quality assurance officers could then review these feedbacks and make judgments regarding performance and standardization. Quality assurance practices could therefore offer objective evaluation metrics as a means to identify major strengths and weaknesses within an SP program, which could then help determine objectives for future areas of development.

The current study has a potential limitation that there was much more data for some health sciences programs than others. Additionally, this is a retrospective single-center study, so these conclusions cannot be generalized, although the current study’s findings could be retested according to other contexts.

Lastly, the present study proposed an example of a logic model based on a synthesis of the current literature aimed at developing a quality management culture in SP programs (Figure 1). In addition to regularly evaluating SP interactions with learners, providing feedback to SPs on maintaining or improving quality, and implementing processes that allow all stakeholders involved in the learning activity to submit feedback, it is also integral to the process of quality improvement that feedback is analyzed and acted upon in a timely manner. Any implemented changes based on feedback should be clearly communicated to stakeholders to ensure congruency and consistency.

## Conclusions

According to the results of the current study, component scoring on SP performance did not discriminate well against all the issues raised; however, qualitative comments did identify certain specific issues related to SP portrayal and the provision of feedback to learners. Additional research is needed to establish standards for continuing SP program quality improvement and for the purposes of evaluating performance consistency, the effectiveness of feedback given to learners, and the reliability of scoring. This represents a gap in the existing literature as the integration of quality assurance practices in routine SP evaluation. To address this, practical guidelines should be created to help define how best to effectively establish and implement a quality improvement culture among SP programs.

## References

[1] Barrows HS (1993). An overview of the uses of standardized patients for teaching and evaluating clinical skills. AAMC. Acad Med.

[2] Plaksin J, Nicholson J, Kundrod S, Zabar S, Kalet A, Altshuler L (2016). The benefits and risks of being a standardized patient: a narrative review of the literature. Patient.

[3] Boulet JR, Smee SM, Dillon GF, Gimpel JR (2009). The use of standardized patient assessments for certification and licensure decisions. Simul Healthc.

[4] Lewis KL, Bohnert CA, Gammon WL (2017). The Association of Standardized Patient Educators (ASPE) Standards of Best Practice (SOBP). Adv Simul (Lond).

[5] Nestel D, Roche J, Battista A (2017). Creating a quality improvement culture in standardized/simulated patient methodology: the role of professional societies. Adv Simul (Lond).

[6] Genereaux M, Nguyen M, Bostwick JR, Vordenberg SE (2021). Using the higher learning commission’s assessment culture matrix to support continuous quality improvement of a simulated patient program. Innov Pharm.

[7] Vordenberg SE, Smith MA, Diez HL, Remington TL, Bostwick JR (2018). Using the Plan-Do-Study-Act (PDSA) model for continuous quality improvement of an established simulated patient program. Innov Pharm.

[8] Furman GE (2008). The role of standardized patient and trainer training in quality assurance for a high-stakes clinical skills examination. Kaohsiung J Med Sci.

[9] Furman GE, Smee S, Wilson C (2010). Quality assurance best practices for simulation-based examinations. Simul Healthc.

[10] Nestel D, Tabak D, Tierney T (2011). Key challenges in simulated patient programs: an international comparative case study. BMC Med Educ.

[11] Makoul G (2006). Commentary: communication skills: how simulation training supplements experiential and humanist learning. Acad Med.

[12] Cantillon P, Stewart B, Haeck K, Bills J, Ker J, Rethans JJ (2010). Simulated patient programmes in Europe: collegiality or separate development?. Med Teach.

[13] Brown CB, Kahraman N (2013). Exploring psychometric models to enhance standardized patient quality assurance: evaluating standardized patient performance over time. Acad Med.

[14] Jamieson S (2004). Likert scales: how to (ab)use them. Med Educ.

[15] Hsieh HF, Shannon SE (2005). Three approaches to qualitative content analysis. Qual Health Res.

[16] Erby LA, Roter DL, Biesecker BB (2011). Examination of standardized patient performance: accuracy and consistency of six standardized patients over time. Patient Educ Couns.

[17] Nestel D, Bearman M (2014). Introduction to simulated patient methodology. Simulated patient methodology: theory, evidence and practice.

[18] Pritchard SA, Denning T, Keating JL, Blackstock FC, Nestel D (2020). "It's not an acting job … don't underestimate what a simulated patient does": a qualitative study exploring the perspectives of simulated patients in health professions education. Simul Healthc.

[19] Nestel D, Tierney T, Kubacki A (2008). Creating authentic simulated patient roles: working with volunteers. Med Educ.

[20] Zhang S, Soreide KK, Kelling SE, Bostwick JR (2018). Quality assurance processes for standardized patient programs. Curr Pharm Teach Learn.

